# Mitochondrial and Y chromosome haplotype motifs as diagnostic markers of Jewish ancestry: a reconsideration

**DOI:** 10.3389/fgene.2014.00384

**Published:** 2014-11-10

**Authors:** Sergio Tofanelli, Luca Taglioli, Stefania Bertoncini, Paolo Francalacci, Anatole Klyosov, Luca Pagani

**Affiliations:** ^1^Laboratorio di Antropologia Molecolare, Dipartimento di Biologia, Università di PisaPisa, Italy; ^2^Dipartimento di Scienze della Natura e del Territorio, Università di SassariSassari, Italy; ^3^The Academy of DNA GenealogyNewton, MA, USA; ^4^Division of Biological Anthropology, University of CambridgeCambridge, UK; ^5^Department of Biological, Geological and Environmental Sciences, University of BolognaBologna, Italy

**Keywords:** haplotype, genetic motifs, NRY, mtDNA, Jewish, Cohanim, Levites, Ashkenazi

## Abstract

Several authors have proposed haplotype motifs based on site variants at the mitochondrial genome (mtDNA) and the non-recombining portion of the Y chromosome (NRY) to trace the genealogies of Jewish people. Here, we analyzed their main approaches and test the feasibility of adopting motifs as ancestry markers through construction of a large database of mtDNA and NRY haplotypes from public genetic genealogical repositories. We verified the reliability of Jewish ancestry prediction based on the Cohen and Levite Modal Haplotypes in their “classical” 6 STR marker format or in the “extended” 12 STR format, as well as four founder mtDNA lineages (HVS-I segments) accounting for about 40% of the current population of Ashkenazi Jews. For this purpose we compared haplotype composition in individuals of self-reported Jewish ancestry with the rest of European, African or Middle Eastern samples, to test for non-random association of ethno-geographic groups and haplotypes. Overall, NRY and mtDNA based motifs, previously reported to differentiate between groups, were found to be more represented in Jewish compared to non-Jewish groups. However, this seems to stem from common ancestors of Jewish lineages being rather recent respect to ancestors of non-Jewish lineages with the same “haplotype signatures.” Moreover, the polyphyly of haplotypes which contain the proposed motifs and the misuse of constant mutation rates heavily affected previous attempts to correctly dating the origin of common ancestries. Accordingly, our results stress the limitations of using the above haplotype motifs as reliable Jewish ancestry predictors and show its inadequacy for forensic or genealogical purposes.

## Introduction

Sequences of allele states or “motifs” based on polymorphisms at the mitochondrial genome (mtDNA) and the non-recombining portion of the Y chromosome (NRY) have been proposed to trace Jewish ancestries. Particularly, Skorecki et al. (1997) first suggested, that the differential distribution of Y-DNA haplotypes based on two markers, the Y Alu polymorphism (YAP) and the Y-STR (Short Tandem Repeat) DYS19, could be a proxy of the patrilineal descent of Cohanim high priests. The YAP-/DYS19 B haplotype was recognized as the possible founding haplotype of the Jewish priesthood. Shortly after that, Thomas et al. (1998) refined this hypothesis on the basis of the variability at 6 Y-SNPs (Single Nucleotide Polymorphisms) and 6 Y-STRs. A modal haplotype was found in Cohen Y chromosomes together with a cluster of closely related haplotypes whether they belonged to Ashkenazim or Sephardic communities, whose coalescence was dated to about the time of the David Kingdom (2619–3221 years ago). Nebel et al. (2000, 2001) defined the Cohen Modal Haplotype or CMH (Table 1) as a 6-locus Y-STR haplotype belonging to haplogroup Eu10, that is J1-M267 according to the current nomenclature (Y-DNA Haplogroup Tree 2014 Version: 9.70). Such motif resulted in 2–3 mutational steps away from other Eu10 modal haplotypes observed in Arabic-speaking groups (MH Galilee, MH Bedouin, MH Palestinians) and one step away from the paraphyletic modal haplotype of the Muslim Kurds belonging to haplogroup Eu9 (J2-M172 in the current nomenclature). In a subsequent study Thomas and colleagues reported CMH Jewish motifs also from a set of South East African haplotypes (Thomas et al., 2000).

In their most recent study on the matter, Hammer et al. (2009) have tried to further investigate the Y-DNA evidence of the biblical descent of Cohanim from a single ancestor (the biblical Aaron) by extending the discrimination power of the CMH from 6- to 12-locus Y-STR haplotypes (eCMH, Table 1). They claimed that the origin of diversity associated to Cohanim J1-P58 chromosomes could be dated between 4280 and 2100 years ago.

**Table 1:** **NRY haplotype motifs**.

	**Haplogroup**	**Tag**	6-locus^*^	12-locus^**^
Ashkenazi Levites	R1a	LMH/eLMH	16 12 25 10 11 13	13 25 16 10 11 14 12 12 10 13 11 30
Cohanim	J1	CMH/eCMH	14 16 23 10 11 12	12 23 14 10 13 15 11 16 12 13 11 30
Israelite & Palestinian	J1	I&A	14 17 22 11 11 12	
Arabs Galilee	J1	GAL	14 17 23 11 11 12	

* *(DYS19, DYS388, DYS390, DYS391, DYS392, DYS393)*.

** *(DYS393, DYS390, DYS19, DYS391, DYS385a, DYS385b, DYS426, DYS388, DYS439, DYS389-I, DYS392, DYS389-II)*.

*eCMH, extended CMH (Cohen Modal Haplotype); eLMH, extended LMH (Levite Modal Haplotype)*.

The Levite Modal Haplotypes (LMH and eLMH, Table 1) were instead proposed by Behar et al. (2003) as alternative Y-STR modal haplotypes within the R1a-M17 haplogroup variation of Ashkenazim Levites, partly shared with the Eastern Europeans. This finding triggered the hypothesis of the origin of Yiddish from a Slavic language (Sorbian) and the introgression of Khazarian Y chromosomes in the initial formation of Ashkenazi Jews some 1000 years ago. However, resequencing analyses found various founder events among Ashkenazi Levites within R1a demonstrating that a particular sub-clade, the M582, would be a signature of a Near Eastern origin in pre-Diaspora times (Rootsi et al., 2013).

Thomas et al. (2002) found signals of much stronger founding events in female-specific (mtDNA) lineages of different Jewish communities than in corresponding male-specific lineages. They found at least 8 modal haplotypes (frequency >10%) at the HVS-I region of the mitochondrial DNA in 8 geographically separated communities of Jews whereas no differentiation was observed at Y-DNA haplotypes of Jewish and host populations.

Using a high-resolution analysis of haplogroups K and N, Behar and his collaborators identified four mtDNA founder lineages as the matrilineal source of about 40% of the current population of Ashkenazi Jews (Behar et al., 2006). Such lineages were described as originating in the Middle East around 2100 years ago “likely from a Hebrew/Levantine mtDNA pool.” However, the complex matrilineal origin of the Ashkenazi seems to have been best represented by Costa et al. (2013), who revealed that the great majority of Ashkenazi maternal lineages were the result of reiterate admixture events within Europe. Concerning potential demographic confounders of the above scenario, Behar et al. (2004) and Guha et al. (2012) have claimed a strong genetic drift to contribute to the unusually high frequency of recessive disease alleles and low mtDNA and Y-DNA diversity in Ashkenazi populations. On the other hand, the recent literature based on genome-wide analyses (Atzmon et al., 2010; Behar et al., 2010; Bray et al., 2010) highlighted the decisive role of admixture in shaping the present Jewish DNA pool.

In the last few years genetic genealogical companies have been recruiting tens of thousands volunteers who accepted to share the results of their genetic testing into public repositories. The largest ancestry database available to date is the Family Tree DNA archive (FtDNA), including more than 506 K records for Y-DNA and about 180 K records for mtDNA divided into nearly 8 K projects, where participants can share their own DNA profile to trace a common heritage by surname, lineage, or geography. Making use of such valuable resource, we constructed a large database of mtDNA and Y-DNA profiles available from >600 FtDNA projects. We explored the database to survey the variability associated to the genetic motifs proposed by the literature in volunteers claiming a Jewish ancestry and in non-Jewish groups of European, African, or Middle Eastern origin.

We aimed at providing an updated experimental background by which to argument faults and pitfalls one may encounter when using haplotype motifs as diagnostic markers of Jewish ancestry.

## Materials and methods

### Databases building

The Y-STR haplotypes and Y-SNPs were downloaded from the Family Tree DNA Y Chromosome Browser (https://www.familytreedna.com/projects.aspx). To match reference motifs (Behar et al., 2003) with standard nomenclature guidelines (Gusmão et al., 2006) allele states at DYS439 were corrected subtracting 4 repeats. The HVS-I haplotypes and mtDNA SNPs were downloaded from the Family Tree DNA mt Chromosome Browser (https://www.familytreedna.com/projects.aspx).

Records (https://www.familytreedna.com/projects.aspx) were first divided in Projects aimed at explicitly reconstructing Jewish ancestry and other Projects. Secondly, we filtered out within and among Projects those records with duplicated kit numbers and uncertain origins. When not available, associations between haplotypes and SNPs were done by kit number. Lastly, we removed haplotypes which did not fit a double criterion: NRY-haplotypes - to be typed at a minimum of 12 Y-STR loci (the set of “extended” motifs) and assigned to haplogroups by either direct SNP typing or upon predictions based on ≥25-locus STR profiles; mtDNA-haplotypes—to be sequenced at least for HVS-I sites 16,024–16,569 and assigned to haplogroups by direct typing of diagnostic SNPs. We obtained a final grid of 62,920 Y-DNA records and 30,469 mtDNA records. Databases were searched for 6 and 12 Y-STR motifs (Jewish and Arabic) and for 4 HVS-I Ashkenazi motifs (Tables 1, 2).

**Table 2:** **mtDNA haplotype motifs**.

	**HG**	**Tag**	**HVR-I**
Ashkenazi	K1a9	K1	16093C, 16224C, 16311C, 16519C, 16524G
	K1a1b1a	K2	16224C, 16234T, 16311C, 16519C
	K2a2a	K3	16224C, 16311C, 16519C
	N1b1	N	16145A, 16176A, 16223T, 16390A, 16519C

One limitation of publicly available genetic genealogical archives is the self-assignment of participants to social/ethnic categories, as is the case of Jewish descent. Another is the putative relatedness among participants within the same ethnic group. While the former limit is hard to ascertain, and sources of error such as adoptions and illegitimate paternities cannot be excluded, by a preliminary analysis close relatedness appears to affect only marginally the summary statistics presented in this paper with respect to those of previous scientific reports. Records sharing a common ancestor were 12 out of 3903 known (0.3%) in Jewish ancestry Projects and 606 out of 48,006 known (1.5%) in the other Projects.

### Calculation of haplotype mutation rates

We employed the following pedigree-based rates:

Average mutation rates per Y-haplotype were obtained according to the “genealogical” method (Klyosov, 2009a; Rozhanskii and Klyosov, 2011) after the calibration for back mutations described therein, and to “germ-line” estimates combining father-to-son pairs data from the literature (29 studies reviewed by Burgarella and Navascue's, 2011, plus Ballantyne et al., 2010).

In the former case the slope of the calibration plot for the 12-locus STRs of eCMH and eLMH motifs fits well to a value of the mutation rate constant of 0.00166 mutation per marker per conditional generation of 25 years, that is 0.020 mutations per haplotype every 25 years.

In the latter case the number of observed mutations in a total of 126,873 meioses (Table S1) gave an average rate 2.113 × 10^−3^ (± 1.369 × 10^−3^) mutations per marker per generation, that is 0.025 ± 0.016 mutations per haplotype per generation. For the trimeric locus DYS426, where no mutations were observed across pedigrees, a regression rate based on a logistic population model was used (0.458 × 10^−3^ mut/gen, Burgarella and Navascue's, 2011).

As average mutation rates per HVSI-haplotype we adopted the pooled pedigree-based rate in Howell et al. (2003) based on 11 studies from the literature: 1.06 × 10^−2^ mut/gen assuming a generation interval of 25 years. It is about seven times higher than the fossil calibrated rate for the 16,051–16,400 segment following Soares et al. (2009): 1.42 × 10^−3^ mut/gen assuming a generation interval of 25 years.

### Network analyses

The use of networks had a dual aim: many of the between-haplotypes intermediate mutational steps, possibly obscured by recurrent mutation or by incomplete sampling, could have been recovered and considered in time estimations; the position of haplotype motifs on the best tree gave a clue of their phylogeny.

Mutational relationships among the Y-DNA 12-locus motifs and HVS-I (16,024–16,569 bp) motifs from individuals of self-reported Jewish descent were visualized by means of the median joining network algorithm implemented in the Network 4.612 software (http://www.fluxus-engineering.com) according to Zalloua et al. (2008). When constructing networks, the default value (10) was given to each HVS-I site and a score calculated upon the variance estimated at each locus was adopted for Y-STR data (score for a given locus = 10 ^*^ total variance over all the loci/variance at that given locus).

For Y-DNA, we selected only haplotypes (*N* = 142) assigned to the R1a1a1-M417 lineage and its downstream subclades containing the LMH motif (16-12-25-10-11-13, Table 1), and haplotypes (*N* = 73) assigned to the J1a2b-P58 lineage and its downstream subclades containing the CMH motif (14-16-23-10-11-12, Table 1). The locus DYS385 was not used because alleles cannot be correctly assigned to the specific duplicated region, and DYS389II was treated as DYS389b = DYS389II - DYS389I. Coalescence times were estimated from networks by the rho statistic using “pedigrees”- as well as “genealogically”- based rates.

For mtDNA, we selected only haplotypes containing the three K motifs (16093C 16224C 16311C 16519C 16524G, motif_K1; 16224C 16234T 16311C 16519C, motif_K2; 16224C 16311C 16519C, motif_K3; Table 2). Coalescence times were estimated from networks by the rho statistic using “pedigrees”- based rates.

### Calculation of bayesian conditional probability of assignation

Bayes theorem was used to calculate the conditional probability of an individual to belong to the Jewish population given that such individuals carries a “diagnostic” NRY or mtDNA motif. Such probability is function of the frequency of a given haplotype in Jews and non-Jews (obtained from Tables 3–**5**) and the proportion of Jewish individuals (estimated to be in the order of 13 Million) in the global population (7.2 Billion people).

**Table 3:** **Distribution of NRY (typed and predicted upon Y-STRs) and mtDNA motifs in the FTDNA public database**.

**Y-DNA**			**Jewish motifs**								**Arab motifs**			
	***N***		**LMH**		**eLMH**		**CMH**		**eCMH**		**GAL**		**I & P**	
	**Typed**	**Predicted**	**Typed**	**Predicted**	**Typed**	**Predicted**	**Typed**	**Predicted**	**Typed**	**Predicted**	**Typed**	**Predicted**	**Typed**	**Predicted**
Jews	5281		155		93		343		81		36		0	
	1103	4178	32	123			270	73	64	17	1	35	0	0
Non-Jews	57,639		807		107		419		17		794		46	
	5593	51,332	98	709	13	94	92	327	5	12	150	644	4	42

**Table:** 

**mtDNA**		**Ashkenazi motifs**				
	***N***	**K1**	**K2**	**K3**	**N**
		16093C	16224C	16224C	16145A
		16224C	16234T	16311C	16176A
		16311C	16311C	16519C	16223T
		16519C	16519C		16390A
		16524G			16519C
Jews	2818	47	74	93	63
Non-Jews	27,651	21	57	686	25

## Results

The analysis of FtDNA records (Tables 3–**5**) confirmed that no genetic motif transmitted along either the maternal or the paternal line is exclusive of Jews. Nevertheless, it was not possible to extend this conclusion to Jewish subgroups such as Levites and Cohanim because this status is rarely self-reported in FtDNA entries. The only exception is the Y chromosome “Cohen Zadokites Project” which joins putative descendants of the Aaron's nephew Zadok. There, indeed, we could check (Table S2) that participants belonged to a total of 6 different haplogroups with J2, not J1, as modal (63%). No 12-locus haplotype was observed to be private to the members of the Project and the J1-eCMH summed to only 4 out of the 59 records with known origin (~8%).

**Figure 1:**
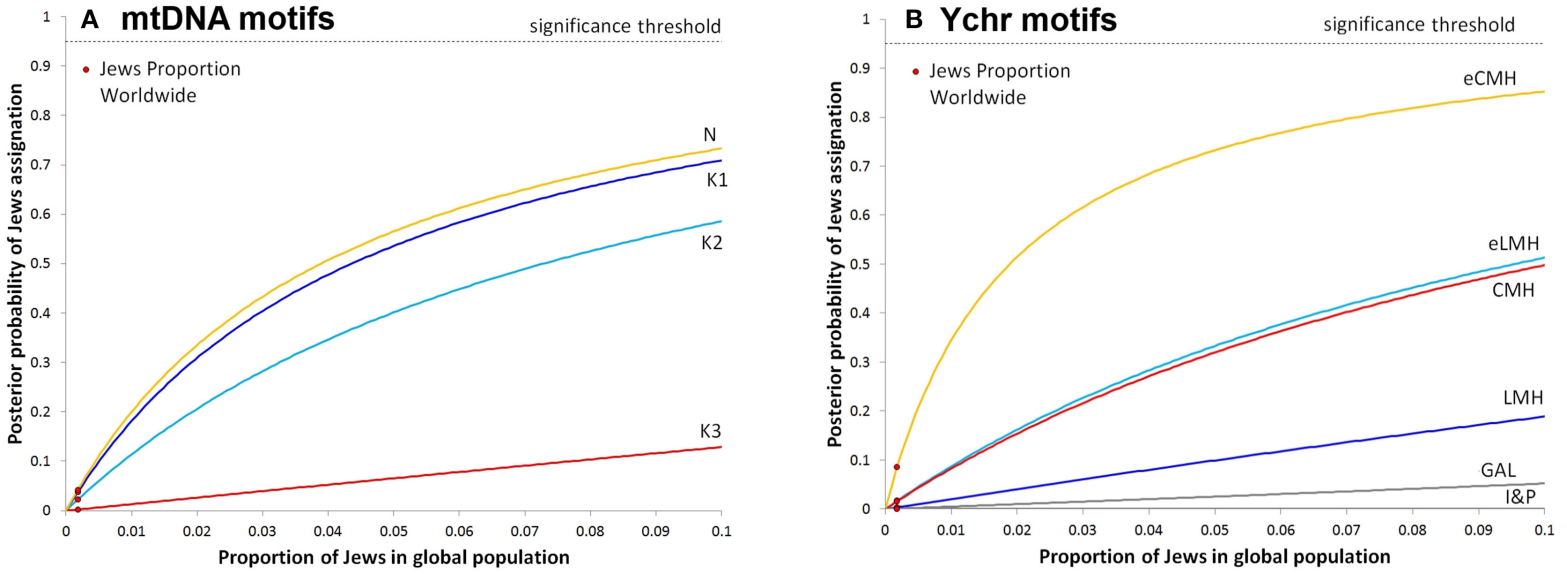
**Estimation of diagnostic power of a set of modal haplotypes to assign a given sample to the Jewish population**. Each line shows the conditional probability (Bayes formula) to assign a given individual carrying mtDNA **(A)** or Ychr **(B)** modal haplotypes to the Jewish population as function of the proportion of Jewish individuals in the sample. Red dots show the obtained posterior probability when inputting the estimated Jewish fraction of the worldwide population.

The analysis of Y haplotype distributions into parental tree branches confirmed that, although there is a clear separation between the distribution of CMH, eCMH, LMH, and eLMH between Jews and non-Jews populations (chi square results in Table 4), no motif is diagnostic of monophyletic haplogroups when the conditional probability of assignment is estimated using the Bayes formula (Figure 1). Even the most resolved Y-DNA motifs (eLMH and eCMH) were found in two or more independent haplogroups whose upper times of divergence are estimated not less than 30 K years ago. This could be explained by a such deep origin and subsequent evolution without any change, by the side-effects of not recognized paternity, gene conversion or, most likely, given the rate of haplotype change (about 24 mutations are expected to occur in 30 K years at extended Y-STR haplotypes) and the high frequency of polyphyletism, by homoplasy.

**Table 4:** **Distribution of motifs in Y-DNA haplogroups (Hg) among 5281 Jews (J) and 57,639 non-Jews (NJ)**.

**Jewish motifs**												**Arab motifs**					
**LMH**			**eLMH**			**CMH**			**eCMH**			**GAL**			**I & P**		
**Hg**	**J**	**NJ**	**Hg**	**J**	**NJ**	**Hg**	**J**	**NJ**	**Hg**	**J**	**NJ**	**Hg**	**J**	**NJ**	**Hg**	**J**	**NJ**
R1a–M198	79	400	R1a–M198/M17/M512	39	36	J–M304	1	1	J1–M267	71	12	J–M304		2	J1–M267		37
R1a–M417	56	234	R1a–M417/458	39	45	J1–M267	196	236	J1–L147	4		J1–M267	24	414	J1–P58		1
R1–Z280		35	R1–Z280		13	J1–L147	13	8	J1–P58	2	2	J1–L147		12	J1–L222		5
R1a–L260		28	R1a–L260		1	J1–P58	16	18	J1–YSC0000234	2	1	J1–P58	10	21	J2–M172		1
R1a–CTS6	8	3	R1a–CTS6	8	2	J1–YSC0000076	6	16	J1–CTS11741		2	J1–YSC0000076	1		J–others		2
R1a–L1029		12	R1a–L342/L657	5	3	J1–YSC0000234	4	4	R1b–P311	2		J1–L222		252			
R1a–L342/L657	5	5	R1a–Z93	1		J1–Z640/Z644	10	24				J2–M172	1	41			
R1a–Z93	1	4	R1a–CTS3402		6	J1–PF4678	1					J2–L26		4			
R1a–SRY10831	1	7	R1a–CTS3412		1	J1–PF4843	1					J2–M410		1			
R1a–Z287	1	4	R1b–L23	1		J1–ZS227	1					J–others		40			
R1a–CTS11962	1	14				J1–F450	2					R1a–M198/M17/M512		1			
R1a–CTS3402	1	19				J1–CTS11741	1	2				R1b–M269		1			
R1a–CTS3412	1	1				J2–M172	67	74				R1b–P311		1			
R1b–M269		1				J2–M67	8	16				R–others		2			
R1b–L23	1					J2–L210	6	8				Others		2			
R1b–P311						J2–L25	1	1									
R–others		35				J2–L26	2										
others		5				J2–Z474	1	1									
						J2–Z482	1										
						J2–M410	1										
						J–others		5									
						R1a–M417/458		1									
						R1b–M269	1										
						R1b–L23	1										
						R1b–P311	2										
						R–others		1									
						Others	1	2									
Total	155	807		93	107		343	419		81	17		36	794		0	46
%	2.9	1.4		1.8	0.2		6.5	0.7		1.5	0.0		0.7	1.4		0.0	0.1
Chi square test (Yates corrected)	144.6			431.2			1343.2			686.2			50.5			2.5	
	d.f. = 6			d.f. = 5			d.f. = 9			d.f. = 3			d.f. = 6			d.f. = 2	
	*p* = 0.000			*p* = 0.000			*p* = 0.000			*p* = 0.000			*p* = 0.000			*p* = 0.289	

**Figure 2:**
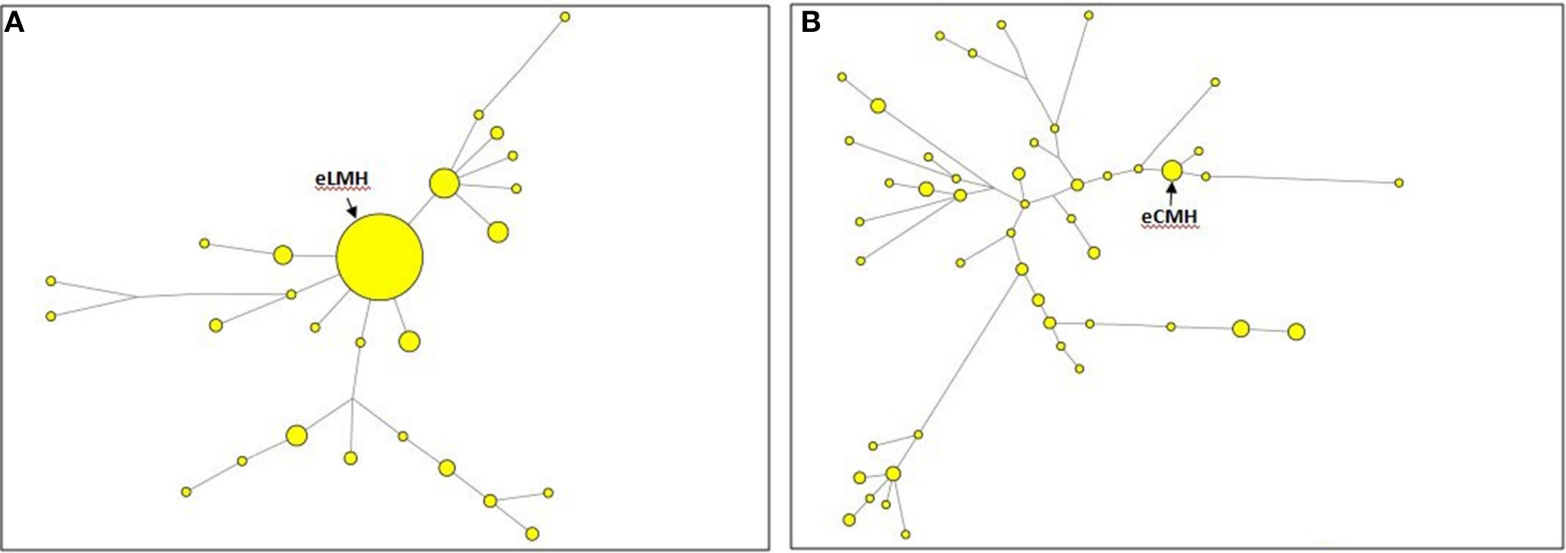
**One of the three most parsimonious trees constructed by the Median-Joining network algorithm**. We used 12-locus Y-STR haplotypes containing the LMH motif assigned to the R1a-M417 lineage **(A)** and the CMH motif assigned to the J1-P58 lineage **(B)** along with their downstream subclades in individuals claiming a Jewish descent. Circles represent haplotypes, with areas proportional to the number of individuals they contain.

It's worth noting that the positions along the trees of eLMH (central, Figure 2A) and eCMH (peripheral, Figure 2B) suggest that the latter might have not been present in the initial pool of founders, but simply be the result of a more recent expansion.

The analysis of the mitochondrial haplotype distributions in Jews indicates that, at least with the current level of SNP resolution, only one motif (K3) out of four has been found in different haplogroups (Table 5).

**Table 5:** **Distribution of the four Ashkenazi mtDNA motifs in haplogroups (Hg) among 2818 Jews (J) and 27,651 non-Jews (NJ)**.

**Ashkenazi motifs**											
**K1**			**K2**			**K3**			**N**		
**Hg**	**J**	**NJ**	**Hg**	**J**	**NJ**	**Hg**	**J**	**NJ**	**Hg**	**J**	**NJ**
K	29	15	K	41	38	K	56	369	N1b	40	18
K1a9	18	6	K1a1b1a	33	17	K1a		2	N1b2	23	7
			K1a3a3		1	K1a1	1	3			
			K2b1		1	K1a14		1			
						K1a16		3			
						K1a19	1	4			
						K1a1a		3			
						K1a1a1		2			
						K1a1a2		4			
						K1a1a2a1		3			
						K1a1b1		4			
						K1a1b1b		5			
						K1a1b1e		4			
						K1a1b2a		1			
						K1a2		2			
						K1a2a		4			
						K1a2b		2			
						K1a2c	1				
						K1a3		4			
						K1a3a		5			
						K1a3a3		3			
						K1a3a4		3			
						K1a4		2			
						K1a4a1	3	22			
						K1a4a1a		3			
						K1a4a1a1		1			
						K1a4a1a3		3			
						K1a4a1a-T195C!		10			
						K1a4a1b		1			
						K1a4a1b1		3			
						K1a4a1c	1	3			
						K1a4a1d		3			
						K1a4a1f	1	3			
						K1a4b		1			
						K1a4d		1			
						K1a-T195C!	1	8			
						K1b1c		1			
						K1b2a		8			
						K1b2a1	1	7			
						K1b2a1a	1	2			
						K1b2a2	1	4			
						K1b2a2a		7			
						K1b2b		12			
						K1c1	1	25			
						K1c1b	1	13			
						K1c1c	2	18			
						K1c1d		3			
						K1c1f		7			
						K2a	1	16			
						K2a10	2	3			
						K2a2a1	18	12			
						K2a3		6			
						K2a5		3			
						K2a5a		1			
						K2a6		28			
						K2a7		3			
						K2a9		3			
						K2b1		9			
Total	47	21		74	57		93	686		63	25
%	0.9	0.0		1.4	0.1		1.8	1.2		1.2	0.0
Chi square test (Yates corrected)	278.2			353.4			97.7			395.0	
	d.f. = 2			d.f. = 3			d.f. = 3			d.f. = 2	
	*p* = 0.000			*p* = 0.000			*p* = 0.000			*p* = 0.000	

On the other hand, the above findings emphasized an over-representation of these motifs in Jews when compared with non-Jews, as well as a significant accumulation of motifs within certain haplogroups (Tables 4, 5). The R1a Jewish haplotypes carrying the Ashkenazi Levite motif LMH (Figure 2A) seem to share the CTS6 variant, whose TMRCA was estimated to be between 1175 ± 341 years ago (using the genealogical rate) and 924 ± 268 years ago (using the germ-line rate). As well, the J1 Jewish haplotypes displaying the Cohanim motif CMH (Figure 2B) seem to share the YSC0000234 variant, whose TMRCA was estimated to be between 1255 ± 441 years ago (using the genealogical rate) and 986 ± 346 years ago (using the germ-line rate). The Jewish haplotypes carrying the K1, K2 and N motifs are exclusive, respectively, of the K1a9, K1a1b1a, and N1b haplogroups (Table 2). Divergence times calculated upon the variability observed at these haplogroups plus the K2a2a1 haplogroups gave recent dates for the common ancestor of all the mtDNA motifs, with those for K2 and K3, respectively, 1370 ± 1241 years ago and 1265 ± 639 years ago, comparable with the times inferred for the common ancestors of the extended Y haplotypes.

## Discussion

Genetic motifs made of sets of non-recombining haploid markers have been long used to trace putative Jewish origins of single individuals or whole populations. Such uni-parental motifs have been also proposed to assign ancestry in association studies and forensic caseworks.

The failure of this practice is inherent to the nature of genetic variation. As a conservative estimate we can expect a novel mutation about every 94 generations (28 substitutions every 2633 transmissions, Howell et al., 2003) in the mitochondrial lineages and about every three generations (3 × 10^−8^ mut/site/gen in deep-rooting pedigrees over 10.15 Mb, Xue et al., 2009) in the NRY lineages.

Therefore, in such genetic systems the larger the number of typed markers, the lower the probability to find out allele sets that exactly match the sequence of the founding ancestors. More specifically, one mutation would occur every 40–50 transmissions at extended Y-STR haplotypes, every 80–100 transmissions at classical 6-locus Y-STR haplotypes and every 140–150 transmissions at the 16,024–16,569 segment of HVS-I haplotypes. Hence, after a few thousand years they all may have mutated more than once.

On the other hand, the lower the number of typed markers the higher the probability of identical haplotypes as result of homoplasy or of an ancient common descent. In other words, the adoption of modal haplotypes as markers for certain historical events must take into account the temporal resolution afforded by the number of variants included in the analyses. Y haplotypes defined by tens of variants minimize the risk of identity by state (IBS), or convergence, therefore allowing for high diagnostic power at the expenses of a shallow temporal resolution (recent TMRCA). Haplotypes defined by 6 or 12 STRs, such as the CMH and LMH or the eCMH and eLMH, enable a deeper temporal resolution, but with an increased error due to recurrent mutations. The adoption of whole mtDNA sequences could improve the resolution of certain maternal phylogenies. As per the many-Y-STR loci, however, this would necessarily affect the trade-off between power to discriminate between IBD and IBS and temporal resolution.

Our results are a demonstration of this argument: the use of non-recombinant haplotype motifs as diagnostic markers of Jewish ancestry was shown to be strongly misleading when not supported by knowledge at more informative regions or whole sequences. Of the motifs previously assumed to trace specific Jewish ancestries, none resulted identical by descent (IBD), that is inherited without modifications from a common ancestor. With few exceptions, motifs, whether from the NRY or the mtDNA, were observed in at least two independent lineages, sometimes belonging to ethnic groups with different cultural or geographic affiliations. To explain the polyphyletic pattern of haplotype distribution across the analyzed groups, we envisage, as the most parsimonious explanation, multiple founder events and/or reshuffling of the genomic pools through the long history of dispersal and admixture of Jewish communities since their foundation.

To make an example, the “CMH” signature, in its classical and extended version, has been observed in many haplotypes of inhabitants of the Arabian Peninsula with typical Arabic names, as well as in many Jewish people belonging to haplogroups J1 and J2. The distribution of CMHs by ethnics and haplogroups is such to suggest that gene conversions, adoptions and illegitimate paternities could affect only marginally the results unless they were multiple and mainly occurred hundreds years ago.

An easier explanation is that, between 7600 and 10,400 years bp (95% CI), the “Cohen Modal Haplotype” was an ancestral haplotype for the historical inhabitants of the Arabian Peninsula. About 4000 ± 520 years ago the establishing Jewish population carried this “modal haplotype” along with the future Arabs, who at that time had a common ancestor with the future Jews (Klyosov, 2010). By around the tenth century AD, a slightly modified “recent CMH” split from the “older CMH” (in more extended haplotype formats), while both of them contained the 6 marker signature of the “CMH,” which is still present in many Arabic haplotypes. This “recent CMH” became the ancestral haplotype for a separate albeit recent Jewish lineage within haplogroup J1. If one consider only “CMH” haplotypes within this population, a common ancestor who lived around 1255-986 years ago can be identified.

Focusing on mitochondrial motifs, the presence of variants at fast mutating sites such as 16,311 and 16,519 increases the potential for the occurrence of recurrent mutations at HVS-I segments. This is particularly critical within haplogroup K. Monophyly and recent TMRCAs, namely genealogies traceable down to a single recent ancestor, could be invoked for K1, K2, and N motifs while admixture and multiple founders should be invoked for the K3 motif in the absence of a better resolution, in line with full genome and re-sequencing data.

An additional element of uncertainty is played by the choice of the mutational rate. Haplotype mutates changing their alleles unpredictably, and only an average number of mutations over a given time can be predicted with a certain probability, based on mutation rate constants and on how “old” is the group of haplotypes in terms of a timespan from their common ancestor.

The issue of which is the most suitable haplotype mutation rate constant to be applied to tracing historical pathways of human populations has been hotly debated (see Soares et al., 2009; Wei et al., 2013) and the recent availability of whole-genome and resequencing data did not solved it. It's widely accepted that mutation counts between diverging haplotypes saturate quite quickly because of recurrent mutations, especially at STR markers. It's also implicit that rates calibrated upon infinite branching models and evolutionary timescales inflate TMRCAs of haplotypes which came to diverged in historical times. It is the case of the rate proposed by Zhivotovsky et al. (2004), which was used by Hammer and coworkers to sustain that the age of eCMHs is compatible with the foundation of Cohanim priesthood (see critique in Klyosov, 2009b). As well, it's apparent that germ-line, fathers-to-sons or deep-rooting pedigrees based estimates are often supported by very poor statistics, the mutations observed at some marker being very few or none at all.

To complicate the picture is the concept that a constant rate does not exist in the real world. By itself, the number of years in generation is a floating value, it depends on cultural habits, religion views, age at childs' birth, nutrition, health and other conditions of life on a given territory at a given time. More, the probability of a novel mutation to appear depends on the structure of the genomic region where it happens and its fate largely depends on the size and demography of the community it belongs.

As a rule, the longer timespan to a common ancestor of a group of haplotypes, the less “diagnostics” a motif and more uncertain time estimates. Only groups with recent common ancestors have rather predictable motifs, as is the case of Ashkenazi Jews at some mtDNA haplogroups and Y-R1a sub-clades expanded in the last thousand years, because not much time left since the common ancestor, and his haplotype is still around having relatively few mutations.

All of the proposed motifs were found to be unevenly distributed across individuals grouped according to their Jewish and not Jewish self-reported ancestry, almost always with a significant enrichment in Jews (Tables 4, 5). However, the heterogeneous composition of haplotypes containing them affected any attempt to correctly dating their origin. Higher resolution SNP typing and, hopefully, the availability of full sequences, might help refining the phylogeny of such markers, ultimately clarifying their role and time from the foundation of the Jewish groups.

In conclusion, while the observed distribution of sub-clades of haplotypes at mitochondrial and Y chromosome non-recombinant genomes might be compatible with founder events in recent times at the origin of Jewish groups as Cohenite, Levite, Ashkenazite, the overall substantial polyphyletism as well as their systematic occurrence in non-Jewish groups highlights the lack of support for using them either as markers of Jewish ancestry or Biblical tales.

### Conflict of interest statement

The authors declare that the research was conducted in the absence of any commercial or financial relationships that could be construed as a potential conflict of interest.
