# Supplementary material for: Mitochondrial and Y chromosome haplotype motifs as diagnostic markers of Jewish ancestry: a reconsideration
Source: Front Genet. 2014 Nov 10;5:384. doi: 10.3389/fgene.2014.00384 (PMC4229899; doi:10.3389/fgene.2014.00384)
Supplement: Supplementary file 1 [file Table1.DOCX]

**Table S1. Combined meiosis data from 30 published studies which have been used to estimate the germ-line mutation rate for 12-locus Y-STR haplotypes.**

| Locus | DYS19 | DYS385a | DYS385b | DYS388 | DYS389I | DYS389II | DYS390 | DYS391 | DYS392 | DYS393 | DYS426 | DYS439 |
| --- | --- | --- | --- | --- | --- | --- | --- | --- | --- | --- | --- | --- |
| Burgarella & Navascue´s 2011 | | | | | | | | | | | | |
| Mutations | 32 | - | - | 1 | 32 | 40 | 30 | 38 | 6 | 32 | - | 51 |
| Meiosis | 14632 | - | - | 2394 | 12651 | 12622 | 14131 | 13995 | 13948 | 14632 | - | 9313 |
| Ballantyne et al. 2011 | | | | | | | | | | | | |
| Mutations | 7 | 3 | 6 | 0 | 9 | 6 | 2 | 5 | 1 | 7 | 0 | 6 |
| Meiosis | 1756 | 1762 | 1615 | 1635 | 1751 | 1743 | 1758 | 1759 | 1728 | 1756 | 1735 | 1736 |
| Combined | | | | | | | | | | | | |
| Mutations | 39 | 3 | 6 | 1 | 41 | 46 | 32 | 43 | 7 | 39 | 0 | 57 |
| Meiosis | 16388 | 1762 | 1615 | 4029 | 14402 | 14365 | 15889 | 15754 | 15676 | 16388 | 1735 | 12667 |
