# Supplementary material for: Mitochondrial and Y chromosome haplotype motifs as diagnostic markers of Jewish ancestry: a reconsideration
Source: Front Genet. 2014 Nov 10;5:384. doi: 10.3389/fgene.2014.00384 (PMC4229899; doi:10.3389/fgene.2014.00384)
Supplement: Supplementary file 2 [file Table2.DOCX]

**Table S2. Assignment of Jewish Y-motifs to haplogroups in participants to the FtDNA Cohen Zadokites Project.**

| Haplogroup | N | CMH | eCMH | LMH | eLMH | I & A | GAL |
| --- | --- | --- | --- | --- | --- | --- | --- |
| J1 | 16 | 11 | 4 |  |  |  |  |
| J2 | 37 | 2 |  |  |  |  |  |
| I | 2 |  |  |  |  |  |  |
| L | 1 |  |  |  |  |  |  |
| R1b | 1 |  |  |  |  |  |  |
| T | 1 |  |  |  |  |  |  |
| unclassified | 1 |  |  |  |  |  |  |
| Total | 59 | 13 | 4 | 0 | 0 | 0 | 0 |
